# The Holistic Impact of Classroom Spaces on Learning in Specific Subjects

**DOI:** 10.1177/0013916516648735

**Published:** 2016-05-16

**Authors:** Peter Barrett, Fay Davies, Yufan Zhang, Lucinda Barrett

**Affiliations:** 1 University of Salford, Salford, UK; 2 University of Huddersfield, West Yorkshire, UK; 3 Nutbox Consultancy, UK

**Keywords:** school design, learning outcomes, reading, writing, math, kindergarten to fifth grade, empirical study, multilevel modeling

## Abstract

The Holistic Evidence and Design (HEAD) study of U.K. primary schools sought to isolate the impact of the physical design of classrooms on the learning progress of pupils aged from 5 to 11 years (U.S. kindergarten to fifth grade). One hundred fifty-three classrooms were assessed and links made to the learning of the 3,766 pupils in them. Through multilevel modeling, the role of physical design was isolated from the influences of the pupils’ characteristics. This article presents analyses for the three main subjects assessed, namely, reading, writing, and math. Variations in the importance of the physical design parameters are revealed for the learning of each subject. In addition to some common factors, such as lighting, a heavy salience for Individualization in relation to math becomes apparent and the importance emerges of Connection for reading and of Links to Nature for writing. Possible explanations are suggested. These results provide a stimulus for additional finesse in practice and for further investigation by researchers.

The Holistic Evidence and Design (HEAD) project investigated how the built environment within and around classrooms in U.K. primary schools influences learning rates in children. Building on a comprehensive Environment-Behavior (E-B) model (Barrett & Barrett, 2010), and informed by work from postoccupancy evaluations (Zhang & Barrett, 2010), analysis of comments from children (Barrett, Zhang, & Barrett, 2011), and teacher opinions (Barrett & Zhang, 2012), a set of propositions was developed to examine how the spaces around us affect learning. The E-B model for the school built environment introduces the concept that learning within spaces is facilitated by the three separate principles of Naturalness, Individualization, and Level of Stimulation. These are derived from consideration of the broad functioning of the brain in tacitly resolving multisensory inputs and relate, respectively, to the natural, personal, and task environments that simultaneously surround a person in a given space.

Within the Naturalness principle, much research has been carried out about optimum: lighting levels (Heschong Mahone Group, 1999), acoustics (Dockrell & Shield, 2006), learning temperatures (NASUWT, 2012), and air quality levels (Clements-Croome, Awbi, Bako-Biro, Kocchar, & Williams, 2008; Mumovic et al., 2009). It is easy to see how each of these fundamental environmental measures could influence the ability of a child to concentrate on learning in a classroom. U.K. school building regulations did include design requirements to create classrooms that meet minimum standards in these key environmental measures (Department for Education and Skills [DfES], 2003). In addition, within the Naturalness principle, a proposition about “Links to Nature” was included as natural elements or views have been shown to improve cognitive function (R. Kaplan & Kaplan, 1989; Tanner, 2009; Wells, 2000).

Beyond these basic “comfort” requirements, to actively learn within an environment, further features are required. It has been shown that to engender the concentration essential for learning, a child needs to be actively engaged with the learning process. Hattie (2008) noted how the effect of pupils’ commitment to their learning can be fostered using pupil-centered strategies. In the E-B model within the Individualization principle, the elements Ownership and Flexibility address how well the classroom is adapted to the child’s viewpoint. Ownership in particular is related to how much the room is individualized for both the class as a whole and for each pupil, with the aim of creating the child-centered environment that is seen to be important for learning (Killeen, Evans, & Danko, 2003; Skinner, Wellborn, & Connell, 1990). Both Ownership and Flexibility are highlighted by Higgins, Hall, Wall, Woolner, and McCaughey (2005) as being important aspects of the physical environment of the classroom. Connection is the third Individualization parameter. It is a measure of the width and orienting features of corridors, so aiding clear navigation, and location, within the school (Alexander, Ishikawa, & Silverstein, 1977; Tanner, 2009).

The third principle of Level of Stimulation aims to create measures that put a scale on the visual stimulation of a classroom. This is done through two measures: Color and Complexity. The scientific research into color is extensive and color can affect children’s moods, mental clarity, and energy levels (Englebrecht, 2003). The measure of Complexity here relates to visual impact from both architectural and display elements in the classroom. For example, Fisher, Godwin, and Seltman (2014) found more distraction and off-task behavior in children in more visually complex environments.

To summarize, the three design principles were split into 10 classroom parameters as follows:

- Naturalness: Light, Sound, Temperature, Air Quality, Links to Nature
- Individualization: Ownership, Flexibility, Connection
- Level of Stimulation: Complexity, Color.

More detailed descriptions of the 10 built environmental parameters of Light, Sound, Temperature, Air Quality, Links to Nature, Ownership, Flexibility, Complexity, and Color are given in Table 1 (Barrett, Davies, Zhang, & Barrett, 2015) and illustrated in Barrett, Zhang, Davies, and Barrett (2015; see Table 1).

**Table 1.** Environment-Behavior Factors Model.

Design principles	Design parameters		Indicators		Factors	Measurement criteria making up high rating
Naturalness	Light	A	The quality and quantity of natural light the classroom can receive	1	Glazing orientation	Larger windows from orientations with no direct sun (glare)
				2	Glazing area/floor area	
		B	The degree to which the lighting level can be controlled	3	Quality of the electrical lighting	Both more and better quality
				4	Shading covering control	Blinds with good functionality/quality
	Sound	C	The frequency of the noise disturbance	5	Noise from the school outside	Large distance from traffic noise or presence of buffer zone
				6	Noise from the school inside	Large distance from playground or busy areas
		D	The degree to which the pupils can hear clearly what the teachers say	7	Length/width	Higher L/W ratio
				8	Carpet area of the room	More coverage is better.
	Temperature	E	The quality and quantity of sun heat the classroom receives	9	Orientation and shading control	Rooms with little sun heat, whether by orientation or shading
		F	The degree to which the central heating system can be controlled	10	Central heating control	Thermostat and radiators in classrooms give better control
	Air quality	G	The degree of respiration that affects the CO_2_ level in a fully occupied classroom	11	Room volume	Greater volume is better.
		H	The degree to which air changes can be adjusted manually	12	Opening window size and position	More opening choices and bigger opening area
				13	MV	MV present
	Links to nature	I	The degree to which the pupils can get access to natural elements	14	Access to nature	Door directly to outside. Plants and wooden chairs/desks in the room.
		J	The degree to which views of nature are available through the window	15	View out	Window sills below child’s eye level and interesting or green near and far views
Individualization	Ownership	K	The degree to which distinct characteristics of the classroom allow a sense of ownership	16	Distinct design features	Originality or novelty character to room. Personalized lockers or coat hooks.
				17	Nature of the display	Child made display
		L	The degree to which the FF&E are comfortable, supporting the learning and teaching	18	Quality of the FF&E	Ergonomic and good quality furniture appropriate for age group
				19	Quality of the chairs and desks	Ergonomic and good quality desks and chairs appropriate for age group
	Flexibility	M	The degree to which the pupils have an appropriate provision of space	20	Classroom floor area and shape: Key Stage appropriate	Larger rooms with simpler shapes for older pupils, but more varied plan shapes for younger pupils
				21	Breakout and storage space attached to the classroom	An attached and dedicated room for breakout and widened corridor for storage
		N	The degree to which the classroom and wall area allows varied learning methods and activities	22	Learning zones: number of zones key stage appropriate	A greater number of well-defined zones for play-based learning, fewer zones, and more formal zones for older pupils
				23	Wall area for display opportunities	Larger is better.
	Connection	O	The presence of a wide pathway and orienting objects with identifiable destinations	24	Corridor width	Wider is better.
				25	Orienting corridor	Displays, landmarks, and daylight with views toward the outside along the pathway
Stimulation	Complexity	P	The degree to which the classroom provides appropriate visual diversity	26	Visual diversity of layout and ceiling	Curvilinear effect: Overall visual complexity including room layout and displays should be balanced; not too high nor too sterile
Appropriate level of		Q	The degree to which the display provide appropriate visual diversity	27	Visual diversity of display	
	Color	R	The degree to which the “color mood” is appropriate for the learning and teaching	28	Wall color and area	Light/white walls with bright highlights or feature wall
				29	Colors of blinds, carpet, chairs, and desks	Bright color works better.
				30	Display color	Bright color works better.

*Note.* MV = mechanical ventilation; FF&E = furniture, fixture, and equipment.

It can be seen that all of the above factors *could* be thought to impact the learning of pupils. In this article, we aim to investigate how the elements of the built classroom environment aid or hinder learning progress for each of three *separate subjects*, namely, reading, writing, and math. Each of these subjects could require different levels of a whole range of pupil skills such as focused concentration, creativity, and problem solving and so may call for different optimal physical learning environments.

Although we focus on the role of the physical features of a large sample of classrooms, we also address pupil characteristics and teacher effects to control out their influences through a range of related measures, twinned with multilevel statistical modeling.

A description of the data collection and research methodology is contained in the next section of this article. This is followed by the results from the subject investigations and then the discussion, and conclusion.

## HEAD Data and Methodology

### Environmental Data

In the U.K. primary school, pupils are based predominantly in one classroom for their learning over a given academic year. U.K. primary school buildings reflect the mild winters and summers that occur. They are heated through the winter season but mostly naturally ventilated in the summer months.

The strategy for the data collection was to achieve a diverse data set, so maximizing the opportunities to explore the impacts of variations in various parameters. Thus, data were collected from 153 classrooms in 27 primary schools in the United Kingdom. The researchers worked with three Local Authority councils (LAs) from three areas with very different characteristics: Blackpool on the coast of northwest England, Hampshire in the south of England, and the outer London borough of Ealing. The building ages and characteristics were chosen to reflect different building regulations from the Victorian era through the 20th century. The Blackpool schools were in a mix of urban and suburban areas with predominantly compact sites having an average site area of 10,600 m^2^; an average total floor area of 3,700 m^2^; and an average pupil count of 400. Three of the Blackpool schools were older and built between 1900 and 1920 and four were modern being built between 1970 and 2006. The Hampshire schools had a mix of rural, suburban, and urban sites, typically with ample outdoor space and recreation areas. They had an average site area of 22,900 m^2^; an average total floor area of 1,840 m^2^; and an average pupil count of 280. One Hampshire school was built in approximately 1880, six were built between 1950 and 1979, and three were built after 1990. The Ealing (outer London) schools were mostly within high population density areas and were large multiform urban schools. They had an average site area of 11,700 m^2^; an average total floor area of 3,050 m^2^; and an average pupil count of 450. Four of the schools were built between 1900 and 1921, three built in the 1950s and 1960s, and three built after 1980. More detail of the building characteristics is given in Barrett, Davies, et al. (2015).

The architectural data collected about the schools consisted of two surveys: a whole school survey taking measures of shared spaces, for example, libraries, assembly halls, gyms, outdoor areas; and a detailed classroom survey for each class. The classroom survey measured both architectural measures such as size of windows, placement of doors, and Interactive whiteboard (IWB), and soft features such as the quality of blinds and number of learning zones. A list of features was assessed in each classroom to provide a database of measurements of the physical environment. Measurement elements were chosen using a hypothesis-led procedure based on the three design principles. It became apparent through preliminary bivariate analyses (see below) that the Level of Stimulation factors were curvilinear so that, for example, with the parameter Complexity neither very sparse/plain rooms nor very cluttered/messy rooms were optimal for learning. Consequently, the scale for Complexity had both very low and very high levels of Complexity scoring low, but intermediate rooms, with a balance of interesting but more ordered features scoring high. Table 1 gives the full details of the E-B model showing how the three principles split into 10 parameters. The table details how the 10 parameters were created from 18 indicators, which were themselves formed from 30 measurement factors. The measurement criteria making up the highest ratings are typified in the last column.

For each of the factors in Table 1, the measures were brought back to a consistent 5-point rating scale. For the curvilinear parameters, the scale was reordered to have a high score in the middle and low scores at either end. As far as possible, measures were based on simple physical measurements, calibrated by the study sample, for example, the areas of windows providing daylighting. For some factors, there was an unavoidable element of researcher subjectivity and this was addressed by separate researchers making individual assessments of the same room and then comparing and establishing a consistent approach. An example of this is (visual) Complexity, where the assessments were a combination of display coverage (some rooms had 100% of their walls covered in displays, some had less than 20%) and a score for how “coherent” or “chaotic” the displays appeared.

Blackpool school and pupil data were collected in the pilot study in the academic year 2011-2012. Hampshire and Ealing data were collected for the academic year 2012-2013. In this study, data from all three areas were combined.

### Pupil Data

Data were collected about each of the 3,766 pupils covering the age range from Year 1 (age 5 years) to Year 6 (age 11 years), and critically, including the classroom in which they resided. The spread of pupils across the three locations was 715 from Blackpool; 1,535 from Hampshire; and 1,480 from Ealing. Fifty percent of the sample were female and the spread across Years 1 to 6 was, respectively, 12% in Year 1, 16% in Year 2, 20% in Year 3, 17% in Year 4, 19% in Year 5, and 16% in Year 6.

We were modeling both pupil-level progress and classroom effects, so it was important to include in the model any pupil factors that could confound the latter effects. Thus, data were collected as to whether the child was in any of the three special categories of having free school meals (FSM—a measure of poverty or socioeconomic status, available to families working less than 16 hr per week with an income, in 2011, for example, under £16,190 per annum), having English as an additional language (EAL in the United Kingdom, but U.S. ESL—English as a second language—used from here in this article), or having special educational needs (SEN; assessed using national criteria). Because of their large impact on educational attainment and linkage to additional funding, data for these three categories are routinely collected for each child in English schools. The representation of these categories in the sample was, respectively, 21%, 23%, and 18%.

Pupil age was another important factor to include as pupils learn at different rates, but in the United Kingdom, their age determines which year group they are in, and pupils in different year groups learn at different rates. In this research, we use both “actual-age” (in months), which determines the pupil’s year group, and “age-in-year” (the number of months the child is past their birthday at the start of the academic year), which is how old each pupil is relative to other pupils in that child’s class.

In educational research, another key predictor of both progress and attainment for each pupil is the level from which that pupil is starting, or start level. In this investigation, the start level is grand-mean-centered on the whole data set to form a pupil variable that is related to how far through the primary school curriculum the pupil is. For the Reading Progress model, this is termed *reading start*. The same is done for the Writing Progress and Math Progress models. A second explanatory variable was calculated from the start grade of each child by relating it to how far ahead or behind a pupil was compared with other pupils in the same year group. The second pupil measure calculated was the start grade group-mean-centered on year group, and for the Reading Progress model, for example, this was termed *reading start-on-age*.

Finally, attendance information was gathered and pupils were removed from the data set if they started in a classroom more than 1 week after the start of the first term. A separate investigation into the attendance rates showed, after removing those children starting late, that progress rates on average did not differ significantly for pupils with poorer attendance. Consequently, attendance rate was not used as a pupil variable in the final model.

The subject progress measure was used as the dependent variable in the regression analyses (reading, writing, or math progress). Thus, data were collected for each pupil for reading, writing, and math improvement over 1 year. For each pupil, the start level at the beginning of the year and the final end level were provided. This was converted to a linear scale using the U.K. National Curriculum (NC) points system, which is widely recognized and is detailed in Barrett, Zhang, Moffat, and Kobbacy (2013). The use of a progress measure rather than final attainment, which is much more common in educational research, is a direct consequence of our hypothesis that the ability of a child to learn in any particular classroom environment is related to aspects of that environment. We were therefore investigating the impact on learning progress for the year that the child spent in that space.

Purely as an aid for interpreting results, there was a final step in creating the pupil measures used in the study. The progress measures (reading progress, writing progress, and math progress) were grand-mean-centered, and the progress measures and each of the pupil variables (subject start-on-age, subject start, actual-age, and age-in-year) were also divided by their respective standard deviations. Each of the variables consequently has a mean of 0 and a standard deviation of 1. This last step “normalizes” the data so that the magnitudes of the calculated regression coefficients are directly comparable with each other, and a coefficient of one indicates a magnitude of 1 standard deviation.

**Figure 1.**
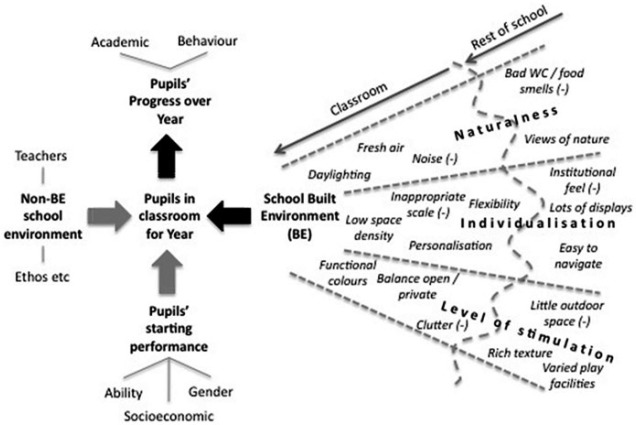
Overview of HEAD research design (with examples of BE factors). *Note.* HEAD = Holistic Evidence and Design; BE = built environment.

In summary, this rich data set concerns both the features of classrooms, *shared* by groups (classes) of pupils, and the *individual* characteristics of the pupils themselves. This creates the conditions to support a type of regression analysis called multilevel analysis, which allows the various influences on the pupils’ academic progress to be isolated. This procedure is detailed in the following section and Figure 1 provides an overview of the research design (see Figure 1).

### Research Methodology

The development of the classroom measures is detailed above. Within the development of the 10 individual classroom parameters, a conscious effort was made to create parameters that were independent of each other. As previous researchers have found (Heschong Mahone Group, 2003; Tanner, 2009), the interaction of various environmental variables makes the link between simple classroom measures and overall comfort levels a nontrivial problem. Therefore, while creating the indicator and factor columns within Table 1, effort was taken to avoid unnecessarily high cross-correlations. This initial process of bivariate analysis is discussed at some length in Barrett, Davies, et al. (2015) and included the check for curvilinear relationships as mentioned above. This procedure led to 10 classroom parameters that were significantly, individually correlated with *overall* progress in learning (the mean of progress in the three subjects). These same 10 parameters were found to have slightly different, but still significant, individual correlations with each of the different subject progress measures (see Table 2). For reading progress correlations with level of Stimulation parameters, Color and Complexity are largest, along with the Connection parameter. Writing progress has highest correlation with Complexity, Links to Nature, and Light. Math progress has highest correlation with Flexibility, Light, and Complexity. The correlations between the subject progress and nine of the classroom parameters are significant at the .01 level.

**Table 2.** Pearson Correlations Between Each of the 10 Environmental Parameters and the Separate Subject Progress Measures.

	Overall progress	Reading progress	Writing progress	Maths progress
Light	.159**	.103**	.137**	.138**
Sound	.042**	.042*	.020	.037*
Temperature	.105**	.085**	.072**	.091**
Air Quality	.122**	.074**	.089**	.127**
Links to Nature	.153**	.107**	.143**	.113**
Ownership	.145**	.120**	.101**	.119**
Flexibility	.153**	.099**	.085**	.180**
Connection	.131**	.132**	.067**	.108**
Complexity	.181**	.130**	.163**	.135**
Color	.177**	.172**	.130**	.113**

* *p* < .05. **p < .01 (two-tailed).

The Pearson correlations between the subject progress measures and the set of pupil explanatory variables are shown in Table 3. The correlations show subject progress is negatively correlated with subject start-on-age and subject start. This describes the fact that as pupils’ start grades are higher, they progress less. This is also shown in the fact that progress is also negatively correlated with actual-age, so that older pupils also progress less than do younger pupils. Gender is not significantly correlated with progress, but pupils who have ESL make better than average progress and pupils with SEN make slower progress. Pupils who have FSM status make significantly less progress in math. These correlations reinforce the importance of including these pupil factors in the analysis alongside the environmental parameters so that the effect of the latter can be isolated.

**Table 3.** Pearson Correlations Between the Subject Progress Measures and Each of the Pupil-Related Explanatory Variables Used in the Model.

Explanatory variable	Reading progress	Explanatory variable	Writing progress	Explanatory variable	Maths progress
Reading start on age	−.153**	Writing start on age	−.184**	Maths start on age	−.135**
Reading start	−.294**	Writing start	−.296**	Math start	−.195**
Age in year	.007		−.016		.003
Actual age	−.229**		−.214**		−.125**
Gender	−.011		.015		−.020
FSM	−.012		−.020		−.060**
ESL	.081**		.097**		.107**
SEN	−.117**		−.105**		−.106**

*Note.* FSM = free school meals; ESL = English as a second language; SEN = special educational needs.

* *p* < .05. **p < .01 (two-tailed).

The second step in the modeling procedure moves on from bivariate analyses, to create a statistical regression model that, in addition to the above pupil measures, links multiple classroom characteristics to the pupil progress measure. Because pupils learn together in classrooms, we could expect the pupil progress between pupils in the same classroom to be more correlated than pupil progress between pupils in different classrooms. For this reason, we used a type of linear regression model that allows data to be clustered in groups, called a multilevel model (MLM). MLM analysis allows modeling of the variance–covariance matrix from the data directly so that the normal requirement of homogeneity of variance across the whole data set can be dropped (Snijders & Bosker, 2012).

The structure of the MLM used for this study was a two-level model where pupils at Level 1 were nested within classrooms at Level 2.^1^

The term *nested* is used as each child learns in only one classroom during the year. MLM analysis also allows unexplained variance to be identified at each of the model levels. Therefore, for example, relevant aspects attaching to the pupils that were not captured by the individual pupil measures used (such as parental education) remain in the unexplained variance at Level 1. Similarly, although we were not explicitly able to model any teacher-related measures, it can be seen that these important elements are compartmented in the unexplained variance at Level 2. A specialist modeling software package MLwiN (Rasbash, Charlton, Browne, Healy, & Cameron, 2009) was used for the study. The modeling building procedure follows that outlined by West, Welch, and Galecki (2007) for a two-level model with clustered data.

A power/error analysis was conducted at the start of the project to estimate what was a sufficient number of classrooms. Maas and Hox (2005) noted that for sample sizes at Level 2 of greater than 50 (we have 157 classrooms), estimates of the regression coefficients, the variance components, and the standard errors were unbiased and accurate. Also multilevel modeling allows within group variance to be more similar than between group variance. The standard errors predicted using a MLM allow for the higher intercorrelation of within group individuals and are consequently much larger than would be predicted using a multiple regression model. Gelman, Hill, and Yajima (2012) noted that MLMs correct for Type 1 errors normally found in multiple correlations problems and also yield more efficient estimates.

After building the levels into the regression model, the explanatory variables were then added. As a test of the efficacy of each additional explanatory variable to improve the model, a likelihood ratio test was carried out. The “−2 × log-likelihood” function was calculated for each of the competing models, that is the simpler model and that with the additional factor. Then, to test whether the latter model was a significant improvement, a comparison was made of the difference in “−2 × log-likelihood” between the two models taking a chi-squared distribution on 1 degree of freedom. This was repeated for each added explanatory variable. The procedure is outlined in Rasbash, Steele, Browne, and Goldstein (2012; Chapter 2).

Following the procedure outlined in West et al. (2007), explanatory variables at Level 1 were added first using a step-up procedure. The two primary predictors of pupil progress that we used in this study were the start grades for each child: subject Start and subject Start-on-age. These two variables were added sequentially and the significance of the model improvement noted using the “−2 × log-likelihood” statistic at each step. The model was then improved by adding random effects on one of the Level 1 variables. The random effects allowed the slope of the regression line to vary for each classroom. The best improvement was found when a random effects variable is added to the subject Start-on-age. The coefficient of the regression line describes the relationship between the average subject progress and the average start level compared with children in the same year. This type of MLM is sometimes called a random slope model (Rasbash et al., 2012).

Each of the other Level 1 explanatory variables were then added to the Level 1 model and the “−2 × log-likelihood” tested to make sure the variables made a significant improvement to the model.

There was deemed to be a significant change where the *p* < .05 (two-tailed). This type of step-up procedure for the model was used because each of the explanatory variables to be added was independent of each other, namely, gender, age, and the key pupil metrics of FSM, ESL, and SEN.

The next part of the process involved adding the classroom explanatory variables at Level 2. Each environmental factor was tested individually by creating a model with just this environmental factor, and there was deemed to be a significant change where the *p* < .05 (two-tailed). Although all were individually significant, the 10 Level 2 variables did exhibit some unavoidable intercorrelations. Because of this what is known as a top-down approach was used when adding these variables where all the 10 parameters are added together. The fitted model therefore showed the combined effect of all these factors, before each factor was removed to test for its individual significance in the overall model (West et al., 2007). As each of the remaining classroom parameters was sequentially removed, the “−2 × log-likelihood” was compared with the full model to see whether there was a significant change (*p* < .10, two-tailed). Where the presence of the parameter significantly improved the model, it was retained; if not, then it was left out. Once all of the parameters that were not significant had been removed, a further procedure was carried out by adding back in each of the rejected parameters. This last step is important as the classroom parameters, because of their intercorrelation, had an impact on each other. A higher *p* value limit was allowed in the final test as both the bivariate analysis and the individual modeling results had already shown the significance of each individual classroom parameter at the higher level.

## Subject Model Results

The results of this series of multilevel modeling exercises for the separate subjects are shown in Table 4, with the “overall” (aggregate of subjects) progress statistics shown for later comparison (Barrett, Davies, et al., 2015; see Table 4).

**Table 4.** Multilevel Modeling Results for Each Subject Model.

	Reading model	Writing model	Maths model	Overall model
Level 1 Factors	Reading Start on Age −0.052 Reading Start −0.318	Writing Start on Age −0.088 Writing Start −0.274	Maths Start on Age −0.016 Maths Start −0.205	Weighted Start on Age 0.090 Weighted Start −0.348
	SEN −0.466 FSM −0.069 Age in Year 0.030	SEN −0.458 FSM −0.123 ESL 0.079 Gender 0.071	SEN −0.333 FSM −0.129 Gender −0.088 ESL 0.084	SEN −0.363 FSM −0.094 ESL 0.086
Level 2 Factors	Light 0.085 Color 0.080 Flexibility 0.076 Complexity 0.062 Connection 0.060	Light 0.111 Flexibility 0.101 LtoN 0.083 Color 0.081 Complexity 0.067	Flexibility 0.197 Light 0.176 Ownership 0.095	Light 0.141 AQ 0.112 Flexibility 0.115 Complexity 0.085 Temp 0.083 Ownership 0.076 Color 0.074
PRV at Level 1	18.6%	16.9%	13.5%	17.8%
PRV at Level 2	18.9%	22.4%	23.4%	26.1%
Model Fit	% Improvement due to classroom parameters = 9.3%	% Improvement due to classroom parameters = 8.4%	% Improvement due to classroom parameters = 11.7%	% Improvement due to classroom parameters = 16%

*Note.* SEN = special educational needs; FSM = free school meals; ESL = English as a second language; PRV = Proportion Reduction in Variance; LtoN = links to nature; AQ = air quality.

For the Level 1 explanatory variables, on average pupils make less progress as their grades improve as all three (reading start, writing start, and math start) have negative correlations with their respective progress. The start-on-age variables are also negatively correlated showing that children who start ahead of their age group also make less progress. For the other pupil-level variables, pupils with SENs and pupils from poorer backgrounds (FSM pupils) both make significantly less progress than average. For reading progress, the older children in their year group make more progress as shown with the positive correlation with age-in-year. Pupils who have ESL/EAL do significantly better in writing and math progress. Girls make more progress in writing and boys make more progress in math.

At Level 2, the classroom parameters significant across all the three subjects were Light and Flexibility. Flexibility had a particularly large correlation with math progress (coefficient = 0.197). The Level of Stimulation factors of Color and Complexity are also both significant in the Reading and Writing Progress models. Connection is significant in the reading progress model, Links-to-Nature is significant in the Writing Progress model, and Ownership is significant in the Math Progress model.

The two-level nature of the MLM allows variability in pupil progress to be divided between the pupil level and the classroom level. The three subject models all start out approximately equally with roughly 67% of the variance allotted to the pupil level and 33% to the classroom level. As described above, explanatory factors are introduced to fit the regression model initially at the pupil level and then at the classroom level. The amount of the variability that is then explained by the significant factors at each level is called the Proportion Reduction in Variance (PRV). Table 4 also shows the PRV at each level for each of the subject models.

For the Reading Progress model, an approximately equal amount of variance is explained by the pupil factors (18.6%) and the classroom factors (18.9%). At the pupil level, some of the factors that are significant are the pupil start grade, whether they have SEN or are registered as having FSM and also if they are old compared with their class (age-in-year). For the Writing and Math Progress models, the PRV explained by the pupil factors is smaller at 16.9% and 13.5%, respectively, and the PRV explained by the classroom factors is larger at 22.4% and 23.4%, respectively.

An improvement statistic can be found by setting all nonclassroom variables in the model at their average value and calculating the classroom effect on the progress measure for each classroom. By comparing the range of the most effective classroom minus the least effective classroom, as a percentage of the total range of the pupil progress score, gives the improvement percentage due to just the classroom parameters. The improvement statistic shows that on average the classroom factors have an effect of approximately 10% of the total variability in pupil progress for each subject (9.3% for reading, 8.4% for writing, and 11.7% for math, see Table 4).

It is interesting to consider the proportion of the modeled impact on pupils’ subject progress, accounted for by each of the environmental classroom parameters, grouped within the three design principles of Naturalness, Individualization, and Level of Stimulation. This is set out in Table 5 and discussed below (see Table 5).

**Table 5.** Proportion of Increase in Pupils’ Subject Progress Accounted for by Each of the Environmental Factors.

Design principle	Environmental parameter	Reading model proportion (%)	Writing model proportion (%)	Maths model proportion (%)	Overall model proportion (%)
Naturalness		23	44	27	49
	Light	23	25	27	21
	Temperature				12
	Air quality				16
	Links-to-Nature		19		
Individualization		38	23	73	28
	Ownership			24	11
	Flexibility	21	23	49	17
	Connection	17			
Stimulation (level of)		39	33	0	23
	Complexity	17	15		12
	Color	22	18		11

## Discussion

At the classroom level, both Light and Flexibility are significant factors in all three subject models. Light has a slightly larger impact on writing progress, and Flexibility has a larger impact on math progress. As discussed in the previous research (Barrett, Davies, et al., 2015), the Light factor is constructed by measures that identify a good amount of natural light, with no glare, and good light control by shading devices and electrical lighting. For the schools studied, large windows orientated without direct sunlight (E, W, NE, NW, and N) had better results than those receiving direct sun (S, SE, and SW). Glare from direct sun is a problem in U.K. schools, especially given the quite general use of interactive white boards with computer projection. These findings resonate with other studies in which good daylighting has been found to be important for learning in schools (Tanner, 2009) and the Heschong Mahone Group (2003), with the latter also finding glare to be the biggest problem in their U.S. schools’ study.

The other ubiquitous factor was Flexibility, which is a measure of how well designed the classroom space is for the particular age of the pupils, whether it has a small group working area and how well the space is designed for storage. For younger pupils, complex room shapes enabled the differentiation into different learning zones and creation of intimate spaces. For older pupils, larger and squarer rooms enabled flexible working for either group work or whole class learning. The optimal transition in reduction in the number of learning zones was found to be gradual as the pupils’ progress through the year groups. This finding confirms Higgins et al.’s (2005) contention that the most successful design elements in classrooms are likely to be elements of flexibility that can adapt to new curriculum demands and new challenges.

In both the Reading and Writing Progress models, but *not* the Math Progress model, the two Level of Stimulation factors (Color and Complexity) were both significant. These two factors relate to the visual environment of the classroom. The color elements were initially rated with pale and white colors rated low and vivid (saturated) colors rated high. However, as already mentioned, wall and display colors were subsequently found to be *curvilinear* meaning that the optimum level for learning was in the middle of the ranges. Read, Sugawara, and Brandt (1999) found in their study of color and space differentiation in learning environments that intermediate values were optimal for child cooperation. In this study for the parameter Color, white- or pale-colored walls with a colored accent wall or panel and brightly colored furniture were found to be optimum for *learning* as well. Color is widely accepted to stimulate the brain, which has consequential effects on moods, mental clarity, and energy levels (Englebrecht, 2003). Küller, Mikellides, and Janssens (2009) also concluded that a moderate use of good color design can improve overall mood and well-being.

For Complexity, the architectural structure, room layout, and wall display were optimal if the overall balance was considered. Again the parameter was found to be *curvilinear* with both low levels of Complexity and high levels of Complexity rated poorly. A room should be distinctive enough to be unique with a reasonable level of well-maintained displays. This finding reinforces Fisher et al.’s (2014) view that rooms that are overly complex are too stimulating and contribute to off-task behavior. Learners with SENs in particular require spaces free from clutter (Martin, 2014) and with lower levels of complexity and color elements (Scott, 2009). Taken together with Color, it seems that a midlevel of stimulation overall supports the alert relaxation needed for effective study via reading and writing.

There are then three factors that each emerges as significant in only one of the individual subject models and not in the other two. In just the Reading Progress model, Connection was found to be significant. The Connection factor was a measure of the width and way finding features of the corridor spaces. A small investigation was done and found that of the 27 schools in the study, 11 of the schools had libraries in corridors or atrium spaces. These have been termed *corridor libraries* and pupils’ reading progress in schools with these was found to be significantly higher, 4.18 NC points compared with 3.90 NC points in other schools (standard error of 0.05 NC points). This seems to explain why wider corridors, which are accounted for in the model, had a positive impact on reading progress. It also implies that “corridor libraries” could be considered as a positive design feature.

In just the Writing Progress model, Links-to-Nature was found to be significant. The measure of Links-to-Nature described in this study is a measure of natural elements in the classroom (wooden furniture and plants), views of nature from the windows, and whether there is direct access to an outdoor learning zone from the classroom. In their classroom studies, Heschong Mahone Group (2003) and Tanner (2009) found that views that included vegetation and objects in the far distance appeared to support better outcomes for student learning in general.

Studies on the effects of nature or natural views on learning have covered many possible influences. S. Kaplan (1995) introduced the idea of natural spaces being a restorative for the fatigue that can be induced by directed attention or concentration with his Attention Restoration Theory. The theory that attention was restored through activity in green spaces was reported in children with attention deficit disorder (ADD) or attention deficit/hyperactivity disorder (ADHD) by Taylor, Kuo, and Sullivan (2001). Natural outdoor spaces have been linked to improved motor abilities (Fjortoft, 2004). Dillon, Craft, and Best (2007) noted that, while inside the classroom was felt to be owned by the teacher, outdoor spaces were owned by the children. Research into home life has shown children who lived nearby nature had lower levels of stress (Wells & Evans, 2003), and girls had greater self-discipline and concentration with more natural views from their homes (Taylor, Kuo, & Sullivan, 2002). The belief that “being in nature” is good for children has led to the development of a range of outdoor or “forest schools” (Borradaile, 2006), where children are taught predominantly in natural settings. There have also been many attempts to link creativity and natural settings. Atchley, Strayer, and Atchley (2012) showed adults performed better on creative tasks after spending an extended time in natural settings. Within a more controlled study, Benfield, Rainbolt, Bell, and Donovan (2015) showed students in classrooms with natural views scored higher than in an otherwise equivalent windowless room on a college writing course. Natural outdoor spaces have also been linked to more creative play (Campbell & Frost, 1985; O’Brien & Murray, 2005).

Within this research, improved levels of progress owing to Links to Nature were only found to be correlated with writing and not with either reading or math. This would indicate that the particular measure of “links-to-nature” developed during this study is possibly more related to the “creative” nature of writing rather than the more general “restorative” effect, described by S. Kaplan (1995), which should have the effect of improving concentration and progress in all subject areas. That said, this is an aspect of the study that has highlighted an effect that deserves further investigation.

In the Math Progress model, Ownership emerges as significant. The Ownership factor measures how well the classroom reflects its use by pupils; does the room have pupils’ work on the walls, do the pupils have their own lockers or coat pegs, and is the furniture ergonomic for the size of the children. It can be seen that all of the factors significant in the Math Progress model (beyond the Light factor) are from the Individualization principle. Individualization is made up of measures for Flexibility and Ownership and was designed to reflect how well the space was adapted for the use of particular children.

Progress in math is associated with factors that are well accepted such as cognitive ability and understanding of the subject, and teacher quality, and availability of appropriate resources. Unlike other subjects, math is also associated with factors that are less well understood, such as societal expectations (Organization for Economic Co-operation and Development, 2015) and test anxiety (Ng & Lee, 2010). Lyons and Beilock (2012) noted that mathematical interventions aimed at reducing anxiety and building confidence could produce better progress than just more time spent on subject learning. Ceci and Williams (2010) have also related the poor performance of women and girls to a lack of confidence and a lack of confident role models. Therefore, our hypothesis is that appropriately designed, child-centered, personalized spaces may have a positive impact on confidence that flows through to improved performance in math, and quite possibly more so for girls. Again the effect is clear, but further investigation is needed to fully understand this finding.

## Conclusion

Pupils from the age of 5 years spend over 6 hr every day of the week within the school environment. Apart from their home, they spend more time in class than anywhere else. Their primary school journey takes them from vulnerable infants to independent learners and thinkers over a period of 6 years. Classrooms need to materially cater to them over this journey. Within the framework of (Level of) Stimulation, Individualization, and Naturalness (the SIN factors), an investigation has been undertaken of how the differing elements of the classroom environment impact on pupils’ learning as measured by progress in reading, writing, and math.

**Figure 2.**
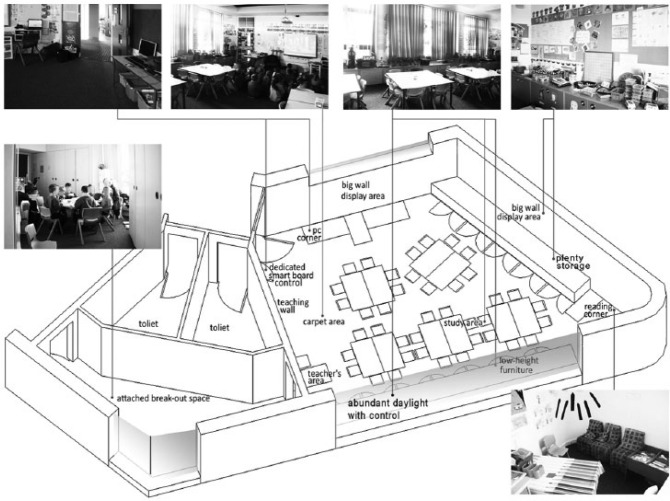
Examples of a range of positive design features for learning.

It is notable that generally, the individual correlations between the 10 design parameters and learning progress are relatively small. It is a feature of this study that it has successfully isolated the influence of these multiple factors in holistic, naturalistic environments and revealed how there are typically multiple factors at play. Figure 2 illustrates how some of these can come together in a typical classroom. It should be clear that the evidence base created through this research raises multiple issues, but resolving these into a coherent design in a particular location still remains a significant design challenge (see Figure 2).

That said, if resolved successfully, the return can be great, as although the individual correlations are small, the combined impacts on learning have been modeled as being substantial. For each of the different subject models, the aspects of the classroom environment taken together explained approximately 10% of the variability in the pupil performance. These are big effects and can be compared with Hattie’s (2008) estimate that “teacher effects” are thought to be of the order of 8% to 20%. Furthermore, as discussed in Barrett, Davies, et al. (2015), their combined effect on “overall” pupil progress is greater still at 16%. This latter measure is highly relevant in the U.K. context, where all of the three subjects discussed above are generally taught in the same classroom and sometimes at the same time.

Although most primary school classrooms will probably remain *the* place where a class learns about all three subjects, the findings of this study highlight some subject-specific variations in the optimal characteristics of the physical space provided, that can be taken into account. Our results show both reading and writing performances are particularly affected by the Level of Stimulation parameters. The biggest impact from classroom design is in math progress, where the Individualization of the classroom to the child appears to be of paramount importance. There could also be possible, tentative, implications for secondary school design, where subject-specialist classrooms are more common.

The focus of the results is on the ambient environmental conditions delivered over a whole year; however, these findings could be used in practice by teachers as they move from one subject to the next and by designers in terms of creating opportunities for dynamically configurable spaces. The factors involved are generally highly practicable to achieve, and in many cases, improvements would not be expensive.

The study by the nature of its design has strengths, such as the large sample size and detailed level of granularity, at the classroom level. But it also has limitations. These relate mainly to practical limits on the collection of data. Information gained was limited to English schools and of course the application of the results beyond this context should be approached with care. There are also data limitations on the multiple aspects known to be important for learning progress (e.g., Hattie, 2008). For example, data could not be accessed on individual pupil characteristics such as socioeconomic background, parental education, and individual mental capabilities. It would be better to have this information in the model, but for this study, they are at least isolated in the multilevel modeling, as unexplained Level 1 (pupil) effects. Within the classroom, measures of teacher quality and pedagogy could not be obtained. In the first case, issues confidentiality proved problematic, as it had been hoped to gain this data. However, the impact of variability in teacher effectiveness is isolated within the unexplained part of the Level 2 (classroom) effects. The impacts of different pedagogical approaches is an area where there is a strong need for further work as an appropriate fit with spatial layout is likely to be influential. In English schools, it appears that quite a consistent “blended approach” is taken, but this is a subjective view, and variations, especially in specialist schools and different countries, deserve careful attention.

From the researchers initial experience, what seems to make the difference to practitioners is *evidence*, and so a raised awareness that the issues identified in this study *do* impact on learning. Until now, most of these issues seem to have occupied a blind spot for practitioners, and so have been unaddressed and unmanaged, hence the variability found in these factors. Once they come into focus, rapid and effective action is entirely possible and likely. For researchers, these broad findings provide a foundation to investigate further the underlying reasons for the identified subject differences so that these can be explored and explained further.

## Notes

1. A three-level model including a “school” level was tested but was found not to improve the explanatory power of the analysis. Reasons for this are discussed in Barrett, Davies, Zhang, and Barrett (2015).

## References

[bibr1-0013916516648735] Alexander C. Ishikawa S. Silverstein M. (1977). A pattern language. New York, NY: Oxford University Press.

[bibr2-0013916516648735] Atchley R. A. Strayer D. L. Atchley P. (2012). Creativity in the wild: Improving creative reasoning through immersion in natural settings. PLoS ONE 7(12), e51474. doi:10.1371/journal.pone.0051474 23251547 PMC3520840

[bibr3-0013916516648735] Barrett P. S. Barrett L. (2010). The potential of positive places: Senses, brain and spaces. Intelligent Buildings International, 2, 218-228. doi:10.3763/inbi.2010.0042

[bibr4-0013916516648735] Barrett P. S. Davies F. Zhang Y. Barrett L. (2015). The impact of classroom design on pupils’ learning: Final results of a holistic, multi-level analysis. Building and Environment, 89, 118-133. doi:10.1016/j.buildenv.2015.02.013

[bibr5-0013916516648735] Barrett P. S. Zhang Y. (2012). Teachers’ views on the designs of their primary schools. Intelligent Buildings International, 4, 89-110. doi:10.1080/17508975.2012.672305

[bibr6-0013916516648735] Barrett P. S. Zhang Y. Barrett L. C. (2011). A child’s eye view of primary school built environments. Intelligent Buildings International, 3, 107-123. doi:10.1080/17508975.2011.582315

[bibr7-0013916516648735] Barrett P. S. Zhang Y. Davies F. Barrett L. (2015). Clever classrooms: Summary report of the HEAD project. Salford, UK: University of Salford.

[bibr8-0013916516648735] Barrett P. S. Zhang Y. Moffat J. Kobbacy K. (2013). A holistic, multi-level analysis identifying the impact of classroom design on pupils’ learning. Building and Environment, 59, 678-689. doi:10.1016/j.buildenv.2012.09.016

[bibr9-0013916516648735] Benfield J. A. Rainbolt G. N. Bell P. A. Donovan G. H. (2015). Classrooms with nature views: Evidence of differing student perceptions and behaviors. Environment and Behavior, 47, 140-157. doi:10.1177/0013916513499583

[bibr10-0013916516648735] Borradaile L. (2006). Forest school Scotland: An evaluation. Report to Forestry Commission Scotland and Forest Education Initiative Scotland. Retrieved from http://bees.bridgend.gov.uk/media/1521/Forest%20Schools%20Scotland%20-%20An%20Evaluation.pdf

[bibr11-0013916516648735] Campbell S. D. Frost J. L. (1985). The effects of playground type on the cognitive and social play behaviors of grade two children. In Frost J. L. Sunderlin S. (Eds.), When children play (pp. 115-120). Wheaton, MD: Association for Childhood Education International.

[bibr12-0013916516648735] Ceci S. Williams W. (2010). The mathematics of sex: How biology and society conspire to limit talented women and girls. Oxford, UK: Oxford University Press.

[bibr13-0013916516648735] Clements-Croome D. J. Awbi H. B. Bako-Biro Z. Kocchar N. Williams M. (2008). Ventilation rates in schools. Building and Environment, 43, 362-367. doi:10.1016/j.buildenv.2006.03.018

[bibr14-0013916516648735] Department for Education and Skills. (2003). Building Bulletin 87-Guidelines for environmental design in schools. London, England: Author.

[bibr15-0013916516648735] Dillon P. Craft A. Best P. (with Rigby A. Simms K.). (2007). Turning peases West inside out: Flexible educational environments for developing possibilities and pedagogies. Sunderland, UK: Creative Partnerships Durham.

[bibr16-0013916516648735] Dockrell J. Shield B. (2006). Acoustical barriers in classrooms: The impact of noise on performance in the classroom. British Educational Research Journal, 32, 509-525. doi:10.1080/01411920600635494

[bibr17-0013916516648735] Englebrecht K. (2003). The impact of color on learning. Retrieved from http://sdpl.coe.uga.edu/HTML/W305.pdf

[bibr18-0013916516648735] Fisher A. Godwin K. Seltman H. (2014). Visual environment, attention allocation, and learning in young children: When too much of a good thing may be bad. Psychological Science, 25, 1362-1370. doi:10.1177/0956797614533801 24855019

[bibr19-0013916516648735] Fjortoft I. (2004). Landscape as playscape: The effects of natural environments on children’s play and motor development. Children, Youth and Environments, 14(2), 21-44.

[bibr20-0013916516648735] Gelman A. Hill J. Yajima M. (2012). Why we (usually) don’t have to worry about multiple comparisons. Journal of Research on Educational Effectiveness, 5, 189-211. doi:10.1080/19345747.2011.618213

[bibr21-0013916516648735] Hattie J. (2008). Visible learning: A synthesis of over 800 meta-analyses relating to achievement. Abingdon, UK: Routledge.

[bibr22-0013916516648735] Heschong Mahone Group. (1999). Daylighting in schools. Fair Oaks, CA: Pacific Gas and Electric.

[bibr23-0013916516648735] Heschong Mahone Group. (2003). Windows and classrooms: A study of student performance and the indoor environment. Fair Oaks: Californian Energy Commission.

[bibr24-0013916516648735] Higgins S. Hall E. Wall K. Woolner P. McCaughey C. (2005). The impact of school environments: A literature review. London, England: Design Council.

[bibr25-0013916516648735] Kaplan R. Kaplan S. (1989). The experience of nature: A psychological perspective. New York, NY: Cambridge University Press.

[bibr26-0013916516648735] Kaplan S. (1995). The restorative benefits of nature: Toward an integrative framework. Environmental Psychology, 15, 169-182. doi:10.1016/0272-4944(95)90001-2

[bibr27-0013916516648735] Killeen J. P. Evans G. W. Danko S. (2003). The role of permanent student artwork in students’ sense of ownership in an elementary school. Environment and Behavior, 35, 250-263. doi:10.1177/0013916502250133

[bibr28-0013916516648735] Küller R. Mikellides B. Janssens J. (2009). Color, arousal, and performance—A comparison of three experiments. COLOR Research and Application, 34, 141-152. doi:10.1002/col.20476

[bibr29-0013916516648735] Lyons I. M. Beilock S. L. (2012). Mathematics anxiety: Separating the math from the anxiety. Cerebral Cortex, 22, 2102-2110. doi:10.1093/cercor/bhr289 22016480

[bibr30-0013916516648735] Maas C. J. M. Hox J. J. (2005). Sufficient sample sizes for multilevel modeling. Methodology, 1(3), 86-92. doi:10.1027/1614-2241.1.3.86

[bibr31-0013916516648735] Martin C. S. (2014). Exploring the impact of the design of the physical classroom environment on young children with autism spectrum disorder (ASD). Journal of Research in Special Educational Needs. Advance online publication. doi:10.1111/1471-3802.12092

[bibr32-0013916516648735] Mumovic D. Palmer J. Davies M. Orme M. Ridley I. Oreszczyn T.. . .Wayd P. (2009). Winter indoor air quality, thermal comfort and acoustic performance of newly built secondary schools in England. Building and Environment, 44, 1466-1477. doi:10.1016/j.buildenv.2008.06.014

[bibr33-0013916516648735] National Association of Schoolmasters Union of Women Teachers (NASUWT). (2012). Campaign on excessive temperatures in the classroom. Retrieved from http://www.nasuwt.org.uk/consum/groups/public/@press/documents/nas_download/nasuwt_009168.pdf

[bibr34-0013916516648735] Ng E. L. Lee K. (2010). Children’s task performance under stress and non-stress conditions: A test of the processing efficiency theory. Cognition & Emotion, 24, 1229-1238. doi:10.1080/02699930903172328

[bibr35-0013916516648735] O’Brien L. Murray R. (2005, Autumn). Forest schools in England and Wales: Woodland space to learn and grow. Environmental Education, pp. 25-27.

[bibr36-0013916516648735] Organization for Economic Co-operation and Development. (2015). Mathematics performance (PISA) (indicator). doi:10.1787/04711c74-en

[bibr37-0013916516648735] Rasbash J. Charlton C. Browne W. J. Healy M. Cameron B. (2009). MLwiN (Version 2.1). Bristol, UK: Centre for Multilevel Modelling, University of Bristol.

[bibr38-0013916516648735] Rasbash J. Steele F. Browne W. J. Goldstein H. (2012). A user’s guide to MLwiN, v2.26. Bristol, UK: Centre for Multilevel Modelling, University of Bristol.

[bibr39-0013916516648735] Read M. A. Sugawara A. I. Brandt J. A. (1999). Impact of space and color in the physical environment on preschool children’s cooperative behavior. Environment and Behavior, 31, 413-427. doi:10.1177/00139169921972173

[bibr40-0013916516648735] Scott I. (2009). Designing learning spaces for children on the autistic spectrum. Good Autism Practice, 10(1), 36-59.

[bibr41-0013916516648735] Skinner E. A. Wellborn J. G. Connell J. P. (1990). What it takes to do well in school and whether I’ve got it: A process model of perceived control and children’s engagement and achievement in school. Journal of Educational Psychology, 82(1), 22-32. doi:10.1037/0022-0663.82.1.22

[bibr42-0013916516648735] Snijders T. A. B. Bosker R. J. (2012). Multilevel analysis: An introduction to basic and advanced multilevel modeling (2nd ed.). London, England: Sage.

[bibr43-0013916516648735] Tanner C. K. (2009). Effects of school design on student outcomes. Journal of Educational Administration, 47, 381-399. doi:10.1108/09578230910955809

[bibr44-0013916516648735] Taylor A. F. Kuo F. E. Sullivan W. C. (2001). Coping with ADD: The surprising connection to green play settings. Environment and Behavior, 33, 54-77. doi:10.1177/00139160121972864

[bibr45-0013916516648735] Taylor A. F. Kuo F. E. Sullivan W. C. (2002). Views of nature and self-discipline: Evidence from inner-city children [Special issue: Environment and Children]. Journal of Environmental Psychology, 22, 49-63. doi:10.1006/jevp.2001.0241

[bibr46-0013916516648735] Wells N. M. (2000). At home with nature: Effects of “greenness” on children’s cognitive functioning. Environment and Behavior, 32, 775-795. doi:10.1177/00139160021972793

[bibr47-0013916516648735] Wells N. M. Evans G. (2003). Nearby nature: A buffer of life stress among rural children. Environment and Behavior, 35, 311-330. doi:10.1177/0013916503035003001

[bibr48-0013916516648735] West B. T. Welch K. B. Galecki A. T. (2007). Linear mixed models A practical guide using statistical software. Boca Raton, FL: Chapman & Hall.

[bibr49-0013916516648735] Zhang Y. Barrett P. S. (2010). Findings from a post-occupancy evaluation in the UK primary schools sector. Facilities, 28, 641-656. doi:10.1108/02632771011083685

